# Assessment of Tools for Marker-Assisted Selection in a Marine Commercial Species: Significant Association between MSTN-1 Gene Polymorphism and Growth Traits

**DOI:** 10.1100/2012/369802

**Published:** 2012-04-30

**Authors:** Irma Sánchez-Ramos, Ismael Cross, Jaroslav Mácha, Gonzalo Martínez-Rodríguez, Vladimir Krylov, Laureana Rebordinos

**Affiliations:** ^1^Laboratorio de Genética, Universidad de Cádiz, Poligono Río San Pedro s/n, 11510 Puerto Real, Spain; ^2^Department of Cell Biology, Faculty of Science, Charles University in Prague, Prague 2, Viničná 7, 12843 Prague, Czech Republic; ^3^Instituto de Ciencias Marinas de Andalucía, Consejo Superior de Investigaciones Científicas, República Saharaui, no. 2, 11510 Puerto Real, Spain

## Abstract

Growth is a priority trait from the point of view of genetic improvement. Molecular markers linked to quantitative trait loci (QTL) have been regarded as useful for marker-assisted selection in complex traits as growth. Polymorphisms have been studied in five candidate genes influencing growth in gilthead seabream (*Sparus aurata*): the growth hormone (*GH*), insulin-like growth factor-1 (*IGF-1*), myostatin (*MSTN-1*), prolactin (*PRL*), and somatolactin (*SL*) genes. Specimens evaluated were from a commercial broodstock comprising 131 breeders (from which 36 males and 44 females contributed to the progeny). In all samples eleven gene fragments, covering more than 13,000 bp, generated by PCR-RFLP, were analyzed; tests were made for significant associations between these markers and growth traits. ANOVA results showed a significant association between *MSTN-1* gene polymorphism and growth traits. Pairwise tests revealed several RFLPs in the *MSTN-1* gene with significant heterogeneity of genotypes among size groups. *PRL* and *MSTN-1* genes presented linkage disequilibrium. The *MSTN-1* gene was mapped in the centromeric region of a medium-size acrocentric chromosome pair.

## 1. Introduction

The value of aquaculture production was estimated at USD 98.4 billion in 2008 and has continued to show strong growth, increasing at an average annual growth rate of 6.2 percent [1]. Among fishes, the gilthead seabream* Sparus aurata*, from the Sparidae family, is one of the most important fish species cultivated in the Mediterranean region, where the main producer countries are Greece, Turkey, and Spain [2]. The strong competition between producer companies and the steep increase in production have caused the selling prices of the product to fall; this in turn has seriously reduced the profit margins of the companies, which in many cases have been squeezed to unsustainable levels. For this reason improvements in the systems of cultivation have become essential, and programmes of improvement by selection must be put into action to reduce production costs. Nevertheless, from cultivated species, only a few of them have ongoing selective breeding programmes. Traditionally, these have been carried out successfully using pedigree information by selecting individuals based on breeding values using an *animal model*. However, information on individual genes with medium or large effects cannot be used in this manner [3]. Molecular markers that directly affect or are linked to quantitative trait loci (QTL) have been regarded as useful for marker-assisted selection (MAS) or gene-assisted selection (GAS) programmes. The allelic variations at these individual genes have a major influence on overall phenotypic expression, and the number of genotypes managed is smaller, so it may be faster and more efficient to implement these improvement programs rather than others under industrial production conditions. Besides, traits as growth, with low heritability and relatively few records per traits measured, are those most benefiting from incorporating marker information [4]. 

Tests of association between candidate gene polymorphisms and quantitative traits have been widely applied in recent years using genetic markers. In that context, PCR-RFLPs (polymerase chain reaction-restriction fragment length polymorphisms) have been demonstrated to be very useful genetic markers for candidate gene studies; such studies have revealed polymorphisms associated with quantitative traits in Atlantic salmon *Salmo salar* [5], oysters *Crassostrea gigas* [6], cattle [7, 8], chickens [9], and sheep [10].

Growth is a priority trait from the point of view of genetic improvement since economic advantages can be gained from shortening the time required for production and improving the rate of feed conversion. Increasing homogeneity in the rate of growth of individuals is another characteristic of interest, since it would reduce the number of gradings to be carried out until harvest size is reached. In this context, the search for candidate genes influencing growth has been a major focus of research, and several candidate genes influencing growth in finfish have been isolated from the genome, and their effects quantified [11]. The growth is a complex trait from the genetic point of view because its genetic control is not well known and there are dozens of candidate genes acting upon it.

The five candidate genes selected for this study were growth hormone (*GH*), insulin-like growth factor-1 (*IGF-1*), myostatin (*MSTN-1*), prolactin (*PRL*), and somatolactin (*SL*) genes. All of them are known to be related to the somatotropic axis or transforming growth factors. Growth hormone (*GH*) plays a major role in stimulating somatic growth primarily through the induction of insulin-like growth factor I (*IGF*) [12]. Myostatin (*MSTN*) or growth differentiation factor 8 gene (*GDF-8*) seems to be a negative regulator of skeletal muscle growth in mammals [13]. There are two genes in gilthead seabream that code for MSTN: *MSTN-1* [14] and *MSTN-2* [15]; *MSTN-1* is expressed mainly in skeletal muscle, at both adult and juvenile stages, and *MSTN-2* is expressed almost exclusively in the central nervous system, at late larval stages [14, 15]. The insulin-like growth factor I gene (*IGF-I*) appears to be linked to nutritional condition, environmental adaptation, embryonic development, and growth regulation of teleosts [16]. Other candidate genes acting upon growth are the prolactin (*PRL*) gene, which performs several functions including mitogenic, somatotropic, and osmoregulatory activities [17] and the somatolactin (*SL*) gene, involved in a variety of physiological functions in teleosts [18].

 Genetic mapping of genes allows a complete identification and the location of a gene and hence can be used in programs of genetic improvement in aquaculture. Physical maps enable the integration of linkage maps and karyotypes and are essential tools for comprehensive comparative genomic studies. In addition, the existence of a well-characterized physical map makes it more feasible to undertake a whole genome sequencing project [19]. Here, we report the localization of *MSTN-1* gene, which presented in this work statistically significant association with growth traits, on *S. aurata* chromosomes.

With the object of gaining new understanding of genes related to growth traits in a marine commercial species, the objectives of the present study were to find and describe polymorphisms in candidate genes from *S. aurata* populations produced under commercial conditions and to test for associations between these polymorphisms and growth traits. Furthermore, the *MSTN-1* gene, whose polymorphism has shown, in this study, a statistically significant correlation with growth traits, was mapped to the chromosomes of this species by means of the tyramide signal amplification (TSA) fluorescence system.

## 2. Materials and Methods

### 2.1. Animals and Phenotypic Traits

**Figure 1:**
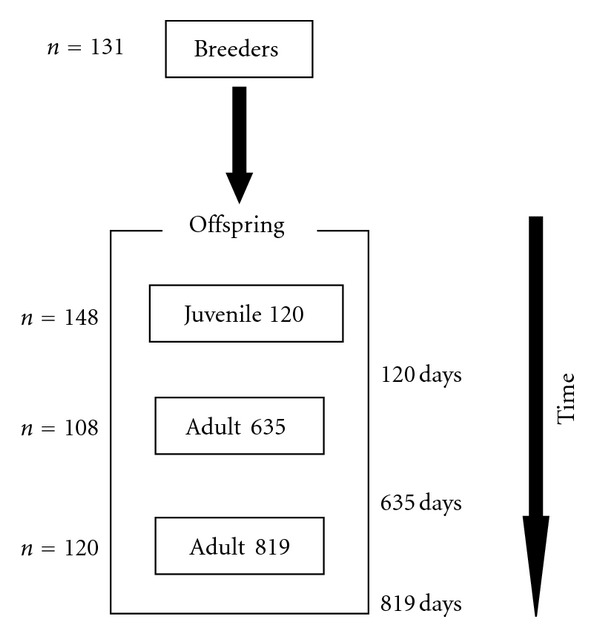
Schematic diagram of sampling during the different life stages.

 The samples were obtained from CUPIMAR S.A., an aquaculture company that operates in the area of the Bay of Cadiz, on the Atlantic coast of southwest Spain. One broodstock and their offspring were analyzed at different stages of production (Figure 1). The broodstock in these installations have mixed origins; wild specimens are added to those from the company's own aquaculture production, because the transformation of males into females after the third year results in a relative lack of males. The broodstock was utilised during several spawning seasons to generate the *F*_1_ that are cultivated and grown for subsequent transfer to the on-growing facilities until reaching harvesting size. The breeders were maintained under environmental conditions of temperature, salinity, and oxygen, according to the management practices of the farm. A total of 131 individual breeders were sampled: 48 males and 83 females (*N*_*B*_ = 131). Within each gender, the breeders were all of similar age.

Fish were anaesthetized with 0.025% 2-phenoxyethanol solution; fork length (*L*) and body weight (*W*) were recorded, and 1–1.5 mL of blood was extracted for DNA isolation. Maturation was induced by control of the photoperiod. Spawning took place in several tanks under production conditions, and offspring were then grouped together in one tank. The offspring originated from the spawning that took place in one single day.

Total residence time, calculated from the hatching date to the first sampling date, ranged between 112 days and 126 days (J120). A total of 148 juvenile fishes (*N*_J120_ = 148) were measured (fork length and body weight) during this period of fourteen days; specimens were graded, and either fin clips were excised or blood samples were taken, in both cases being preserved in 70% ethanol at 4°C till processing for DNA isolation. Juveniles were kept in nursery tanks and transferred to floating cages, where they were cultivated until reaching adult stage. At this stage, the individuals from two cages were sampled at different dates, representing either 635 days (*N*_A635_ = 108) or 819 days (*N*_A819_ = 120) after the date of spawning, respectively, and the 228 individuals in total were measured for fork length and body weight (Figure 1). Finally, quantities of 1.0 to 1.5 mL of blood were extracted for subsequent DNA analysis. A condition index was used to assess relative fatness and provide an estimate of the energy reserves, morphology, and health of the fish and is the parameter that best reflects the criterion utilised by the producer for its commercial classification of the product. Body weight × fork length^−3^  ×  100 is the metric of the condition index (*C*).

### 2.2. DNA Extraction, Genotyping, and Parental Assignment

 Genomic DNA from breeders, adults, and some juveniles was extracted from blood according to the protocol described by Martínez et al. [21]. For the rest of the juveniles, genomic DNA was extracted from fin clips using a modification of the salting-out extraction method. In all cases, the DNA was kept at 4°C until utilisation. All 376 offspring and 131 breeders were genetically characterised using microsatellite markers (multiplex) and familial assignments, determined according to Porta et al. [22].

### 2.3. Polymerase Chain Reaction (PCR) and Sequencing

**Figure 2:**
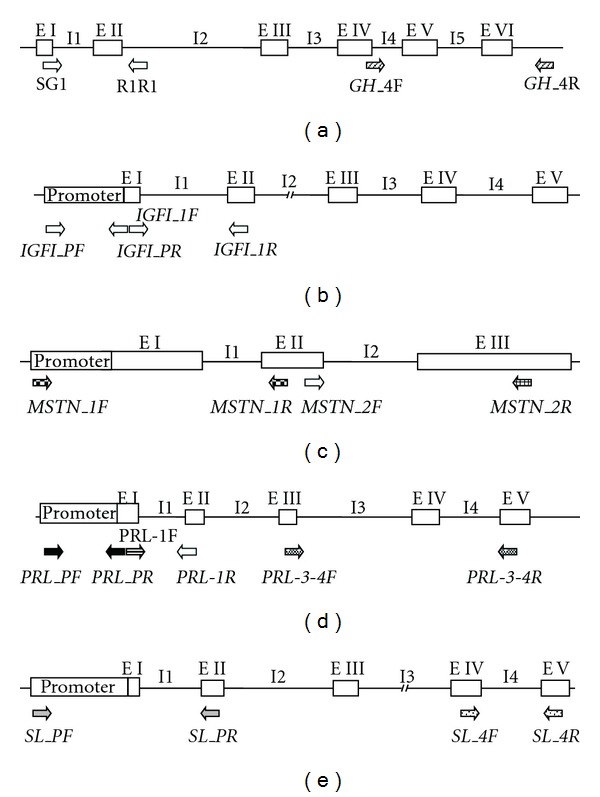
Structure of the candidate genes and oligonucleotide primers used for amplification by PCR in this study. Growth hormone (a), Insulin-Like Growth Factor I (b), Myostatin-1 (c), Prolactin (d), and Somatolactin (e) genes. I: Introns; E: Exons; Arrows indicate relative position of the primers.

 The 11 regions from the five candidate genes that were screened by restriction fragment length polymorphisms (RFLP) are listed in Table 1. Except for growth hormone intron I amplification, which was carried out using oligonucleotides as described in Almuly et al. [20], all other amplification products were performed using forward and reverse primers based on *S. aurata* sequences from GenBank and designed with *Primer3* software [23]. Primers were designed to anneal in the exons and/or promoters of the candidate genes so that we could amplify 0.7–1.9 kb DNA regions including at least one intron or part of the promoter (Table 2 and Figure 2).

The PCR amplification reactions were performed in a Gene Amp PCR System 2700 (Applied Biosystem) thermal cycler that was programmed for denaturation at 94°C for 5 min.; 35 cycles of amplification with 30 s at 94°C, 30 s at 59–63°C (depending on DNA fragment), 1 min. at 72°C; and a final extension at 72°C for 10 min. Reactions were carried out in a final volume of 50 *μ*L with 200 mM dNTPs, 0.2 *μ*M of the forward and reverse primers, 2.0-3.0 mM MgCl_2_, 60–120 ng gilthead seabream genomic DNA, 2–5 U of EuroTaq polymerase (EuroClone Genomics), and 5.0 *μ*L 10x reaction buffer (EuroClone Genomics). The PCR products were examined by electrophoresis through a 1.5% agarose gel containing 0.5 *μ*g mL^−1^ ethidium bromide. Amplified products of the predicted size were cut and purified from gels with a nucleoSpin extract II kit (Macherey-Nagel) before carrying out the sequencing reactions. DNA sequencing was performed with fluorescence-labelled terminator (BigDye Terminator 3.1 Cycle Sequencing Kit; Applied Biosystems) in an ABI3100 Genetic Analyzer. Sequence data from this paper have been deposited with the GenBank Data Library under Accession numbers FJ827497 to FJ827506.

### 2.4. RFLP Detection and Genotyping (PCR-RFLP Alleles)

 The selection of the restriction enzymes needed to observe the RFLPs was performed using software available on the Internet, such as *In silico* [24] and *Webcutter 2.0* (http://rna.lundberg.gu.se/cutter2/). Preferential selection was made for those enzymes that produced cuts within the target sequence and with the number of targets not higher than three, to avoid products of small size that might not be detected later in the electrophoresis. Different enzymes were tested for the different PCR products, with the aim of obtaining at least two RFLPs for each sequence amplified.

 The digestions were performed by incubation of the samples in a water bath at the optimum temperature for each enzyme according to the manufacturer. The reactions were carried out in a final volume of 50 *μ*L containing 0.05–1.0 *μ*L of enzyme (Roche Molecular Biochemicals, Amersham Biosciences or Fermentas), 1x of the appropriate buffer, and 2–5 *μ*L of the PCR product.

To determine the size of the products, the samples were loaded into agarose gels (1–1.5%) in 0.5x TBE with ethidium bromide (0.5 *μ*g mL^−1^). Gels were visualised and analyzed using *Gel Doc XR* Molecular Imager (BioRad) and Quantity One 1-D Analysis programme, respectively.

### 2.5. Statistical Analysis

One-way analysis of variance (ANOVA) was used to test significant associations between the marker genotype (at each of the eight RFLP loci) and the three phenotypic traits (*L*, *W*, and *C*). The procedures utilised in the comparison of means are not very sensitive to the lack of normality. When there are no atypical observations and the sample distributions are approximately symmetric, ANOVA can safely be used even for very few samples (*n* = 4-5) [25]. On the other hand, the results of the *F* test of the ANOVA can be considered reliable, when the largest standard deviation is less than twice as large as the smallest standard deviation [25]. The ANOVA test was applied only in those cases in which the standard deviations complied with this ratio. In those cases in which there were significant values and the sample standard deviations did not comply with this ratio, the nonparametric test of Kruskal-Wallis was applied.

As the gilthead seabream is a protandrous hermaphroditic teleost, in which sex is reversed in males after about 2 years of age, the broodstock presented a marked difference between the means of the males and females. For this reason, we analyzed length, body weight, and condition index variables for each locus by fitting a general linear model (GLM) to the data from breeders that included gender as a factor:

(1) $$Y_{ij}=\mu +\alpha_{i}+\beta_{j}+\varepsilon_{ij},$$

where *Y*_*ij*_ is the phenotypic trait (e.g., weight in breeder group), *μ* is the mean value of the trait, *α*_*i*_ is the genotype at the RFLP marker, *β*_*j*_ is gender, and *ε*_*ij*_ is the random residual.

The proportion of the phenotypic variation explained by each RFLP marker was calculated as *r* ^2^ = SS effect/SS total.

All these tests were applied using the SPSS 14.0 statistical software programme. In all cases when multiple comparisons were made, the Bonferroni adjustment was applied [26].

To determine the existence of linkage disequilibrium between pairs of loci analyzed, the null hypothesis “*genotypes at one locus are independent from genotypes at the other locus*” was tested using the GENEPOP programme package [27]. Contingency tables were created for all pairs of loci in each age group and across all the population, and Fisher's exact test was performed for each table.

 The distribution of the genotypes in each age group, sub-classified by sizes, was studied. The null hypothesis tested was “*the genotypic distribution is identical across the size-group”*. For each locus, the test was performed on contingency tables. An unbiased estimate of the *P* value of a log-likelihood (G)-based exact test was performed [28] using the GENEPOP program package [27]. The test was performed for all pairs of samples, for all loci. According to fork length and body weight distribution, adults and breeders were classified in two groups: small and large (adults) and males and females (breeders). Juveniles were classified in two size groups, small (J-S) and large (J-L) which were subclassified in small (S), medium (M), and large (L). When multiple comparisons were made, the Bonferroni correction was applied to adjust the *α* value for each age group.

### 2.6. Cytogenetic Techniques

Chromosome preparations were made from 1-2-day-old *S. aurata* larvae according to Cross et al. [29]. To make the myostatin probe, first-strand cDNA was obtained from *S. aurata* larvae RNA using a SMART RACE cDNA Amplification Kit (Clontech Laboratories). The cDNA for the *MSTN-1* probe was obtained by PCR from the first-strand cDNA using a forward primer in exon 1: 5′-AGAAGACACGGAGCTGTGC-3′ and a reverse primer in exon 3: 5′-AAGAGCATCCACAACGGTCT-3′. The amplification yielded a fragment of 1025 bp, and its structure was verified by sequencing. Digoxigenin (dig-11-dUTP) labelling of 1 *μ*g DNA probe was performed by random priming using the Decalabel DNA Labeling Kit (Fermentas) according to the manufacturer's instructions. Following the labelling, the sample was purified on a column of QIAquick Gel Extraction Kit (Qiagen) in a final volume of 50 *μ*L. FISH-TSA were carried out essentially according to Krylov et al. [30] with minor modifications. Photographs were taken using a fluorescence microscope (Olympus BX-40) and a SONY EXwave HAD black and white camera. Images were processed and coloured using the ACC Program, v.5.0 (SOFO, Brno, Czech Republic).

## 3. Results

### 3.1. Biometric Data of Breeders and Progeny

The analysis of parentage revealed that a total of 80 fishes, 47 males and 33 females, from 131 breeders, contributed to the progeny (exclusion power > 0.999 and an estimated probability of identity of 2.13 · 10^−31^). A total of 108 families of full and half-siblings, were represented in the sample of 376 offspring samples, with an average of 3.2 (±3.15) descendents per full siblings family.

Table 3 summarizes biometric data (weight, length, and condition index) from breeder and offspring in the different sampled groups. Because of the biology of *S. aurata*, a clear size difference can be observed between male and female breeders; all individuals are males at the end of their first year of life, and at approximately two years of age, most individuals become females. In our study, all the offspring sampled were male. In the breeder group, the 83 females sampled presented mean weight and length values higher than those of the 48 males. The coefficients of variation (CV) can be deduced from Table 3, and for weight, CV is approximately 3 times the values obtained for *L* and for *C*.

### 3.2. Polymorphisms of the Five Candidate Genes

We studied five candidate genes for growth rate in *S. aurata* and tested for association with three quantitative traits (body weight, fork length, and condition factor) at different stages of development: breeders, juveniles, and adults. To find usable RFLP markers in the proposed candidate genes, 11 gene fragments, covering more than 13000 bp, were amplified and sequenced. Several enzymes were chosen to test for usable polymorphism. Four of the 11 fragments studied (*GH_B, IGF-I_B, SL_A*, and *SL_B*) were nonpolymorphic, but seven exhibited polymorphism of size and/or restriction sites, as shown in Table 1 (RFLP patterns obtained in *PRL 3_4* for *Hae*III and *Fok*I were identical, and therefore only one of them (*Fok*I) was considered in the ANOVA analysis).

### 3.3. One-Tail ANOVA Analysis of the Association between Genotypes of the Different Age-Groups, of the Five Candidate Genes and Phenotypic Traits

When the ANOVA test was applied, several putative associations between phenotypic traits (*W*, *L*, and *C*) and genetic marker genotypes were found (Table 4). However, as we tested seven independent hypotheses (seven RFLPs) in each age group the corrected value for *α* was considerably smaller (*α*_Bonferroni_ = 0.05/7 = 0.00714). Under this *α*-correction for the ANOVA test, six putative associations between markers and traits remained significant: five of the six were associated with the *MSTN1* RFLP (in some of the breeders, adults and juvenile groups) (Table 4). After Bonferroni corrections in the Kruskal-Wallis nonparametric tests (applied to *W* and *C* in the juveniles group, and *C* in the A635 adults), two putative *MSTN1*-RFLP associations (both in breeders for *W* and *L*) remained significant (*MSTN1*_*A*-*Hae*III). This marker accounted for 5.8% and 6.2% of the phenotypic variance in *W*-*B* and *L*-*B*, respectively.

Size differences between males and females are a characteristic of this species due to its protrandrous hermaphroditism, by which individuals mature first as males and later change to females. Because of this difference, the study of associations between genotypes and quantitative characteristics in breeders may be affected by sex. When a GLM was used to test for association between genotypic marker and the three quantitative traits (*W*, *L* and *C*) in breeders with gender factor (data not shown), the association between *MSTN1*_*A*-*Hae*III and weight/length traits did not remain significant (*P*_*W*_ = 0.084 and *P*_*L*_ = 0.061).

### 3.4. Pairwise Analysis of the Association between Genotypes, Five Candidate Genes, and Size Classes in Each Age Group

To determine the distribution of genotypes between pairs of size classes for each age group, an analysis using contingency tables was made. Pairwise tests revealed several RFLPs with significant heterogeneity of genotypes among size-group pairs in juveniles, again most of them at the *MSTN-1* gene (*P* ranged from 0.002 to 0.017), and one significant *P* value (*P* = 0.019) in the A819 adult group at the *IGF-I* gene (Table 5). As numerous comparisons were performed for each age group, Bonferroni's correction was applied. Under these corrected values of *α*, the comparison of the small-large groups (S-L), in the RFLP *MSTN1_B*-*Hae*III in the J-S group, remained significant. With this new distribution of individuals, in function of their size, an ANOVA analysis was performed for the small juveniles (S-J120); it was found that, for the weight, there was a significant association (*P* = 0.003) with the RFLP *MSTN1*_*B*-*Hae*III, confirming the putative relationship of *MSTN-1* with growth traits obtained in the various statistical treatments reflected in Tables 4 and 5.

To assess whether heterozygosity at the *MSTN-1* gene influences any of the traits studied, the genotypes were grouped by homozygous/heterozygous type, and this new factor was used in an analysis of variance (data not shown). It was observed that again, for weight of both breeders (*MSTN1_A-Hae*III) and juveniles (*MSTN1*_*B-Hae*III), the homozygous genotypes were larger (*P* < 0.05) than the heterozygous.

When we tested for the existence of linkage disequilibrium between pairs of loci, our results revealed that, curiously, *MSTN1*_*A*-*Dra*I were in linkage disequilibrium with both *PRL-1*-*BspLU11*I (*P* ≤ 0.05) and *PRL-1*-*Eco*RV (*P* ≤ 0.05) (Fisher's exact test), which implies a statistically significant linkage disequilibrium between these two genes, *MSTN1* and *PRL*.

### 3.5. Chromosome Location of the MSTN-1 Gene

**Figure 3:**
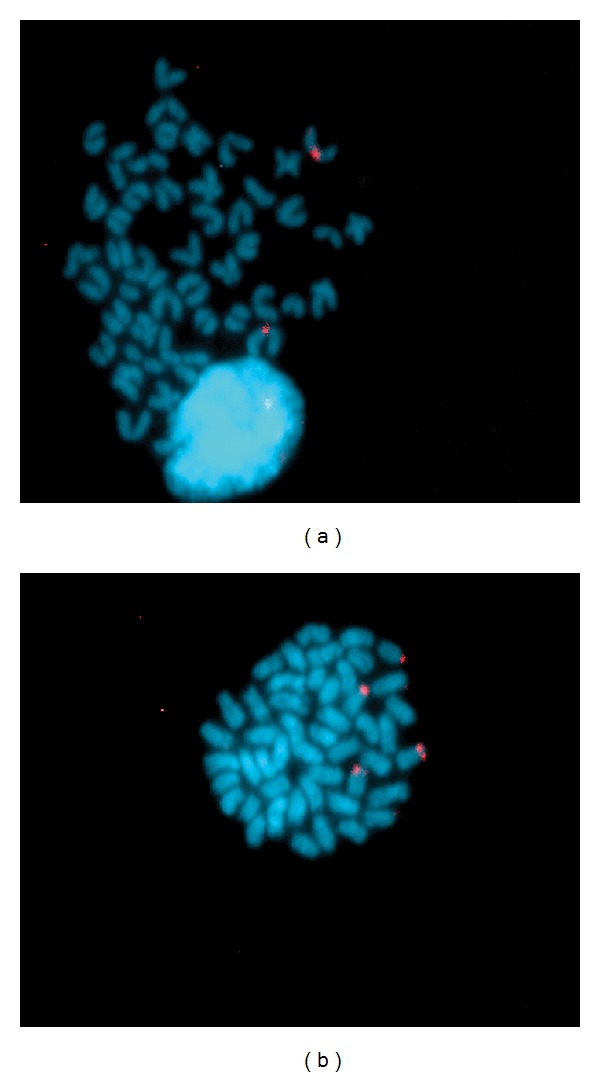
Localization of the myostatin-1 probe on *S. aurata* chromosomes. (a) *MSTN-1* probe in the centromeric region of acrocentric chromosomes. (b) Localization of *MSTN-1* and putative *MSTN-2* genes both in centromeric regions of two different acrocentric chromosomes.

FISH-TSA experiments on *S. aurata* chromosomes have led us to localize the *MSTN-1* gene of *S. aurata* in the centromeric region of two medium-sized acrocentric chromosomes (Figure 3(a)). Signal position was evaluated in 194 metaphase spreads, of which 65.9% displayed a specific signal. The remaining metaphase spreads did not show any FISH signal, apart from a few metaphase spreads (8.7%) that presented four possible signals; that second signal also appears in a centromeric region (Figure 3(b)).

## 4. Discussion

 The infinitesimal model, which assumes that for any particular quantitative trait the individual effects of genes are unknown and usually are only small in their magnitude, does not fully explain the observed patterns of genetic variance. On the contrary, for many traits, allelic variation at individual genes has been shown to have a major influence on overall phenotypic expression [11]. Consequently, there has been substantial investment in livestock sciences directed towards the detection of major/candidate genes differentially influencing trait expression. Taking these factors into account, this study was conducted to obtain more practical results that are closely related to conditions in fish farming and to the biology of the species. The analysis of polymorphism in eleven DNA fragments, belonging to five candidate genes, allowed us to use seven PCR-RFLPs to test for association with three quantitative traits (*W*, *L*, and *C*). The majority of the associations found, after several corrections and nonparametric tests (when necessary), were associated with the *MSTN-1* gene. In breeders, when ANOVAs were performed considering gender as a factor (data not shown), no significant values were obtained. The considerable weight lost at spawning in females may explain the effect of transition from male to female on length and weight traits. Pairwise tests revealed significant heterogeneity of genotypes among size groups in one RFLP marker (*MSTN1*_*B*-*Hae*III) (Table 5) and confirmed the putative relationship of *MSTN-1* with growth traits observed in the ANOVA tests. When we tested for association between homozygous/heterozygous genotypes of the *MSTN-1* gene and phenotype traits, the results suggest selection at the genotype level, with homozygotes being favoured at certain ages for certain alleles present in the population. In order to test rigorously and confirm the putative associations uncovered in this study, cross-test family-based analysis should be applied, under similar conditions of industrial production.

In this study, myostatin polymorphisms were putatively linked with growth traits in *S. aurata* under industrial production conditions. An association between this candidate gene and growth is not surprising because myostatin acts as a negative regulator of skeletal muscle growth [31], and many studies show that myostatin can affect muscle mass in several species [32–34], and the presence in the population of favourable alleles would result in greater growth of those individuals that were homozygous for growth-increasing alleles. Unlike terrestrial vertebrates, piscine MSTN mRNA has been detected in several tissues including muscle, gill, skin, brain, renal, and gonadal tissue [35]; this pattern of expression suggests that MSTN is involved in numerous physiological activities such as osmoregulation, gonadal development, growth, and maturation.

 No significant correlations of molecular variants and quantitative traits were revealed in the present study at the other candidate genes analyzed. However, genes within the somatotropic axis and transforming growth factor superfamily have been shown to influence growth variation in terrestrial livestock, as well as in other vertebrates [11].

We tested the null hypothesis that genotypes at one locus are independent of genotypes at the other locus, for each locus pair across the population. A locus pair located at the same gene (e.g., introns) should be in linkage disequilibrium; the results show a significant linkage disequilibrium between *MSTN-1* and *PRL*. However, not all of them revealed this expected disequilibrium across all age groups. This is because several or many families were present in each group, and different genotypic combinations could be observed. The finding is remarkable because it indicates that genotypes of two RFLP markers (located in *MSTN-1* and *PRL* genes) are not independent. If these loci are located on the same chromosome, they can be selected at the same time. If they are not linked, it means that some genotypic combinations of the two loci are favourable (or are selected against) under some specific conditions and they can be used in marker-assisted selection (MAS), too. Sarropoulou et al. [36] localized the *S. aurata* growth hormone and prolactin genes on radiation hybrid group RH24 of the gene-based radiation hybrid map of gilthead seabream. This location is very interesting because our results suggest linkage disequilibrium between *PRL* and *MSTN-1* genes; therefore, if this disequilibrium is because the two genes are linked in the genome, a linkage between three candidate genes, *PRL*, *MSTN-1*, and *GH*, for putative growth-related QTLs would have been revealed.

In relation to cytogenetic mapping, the application of fluorescence *in situ* hybridization (FISH) to fish genetics and genomics has been widely tested (reviewed in [37]). Chromosome mapping of single copy genes is useful in isolating QTL of importance in aquaculture, and it is the first step towards linking physical and linkage maps. In fishes, to date, only one study, on *D. rerio*, has been found in which the *MSTN-1* gene was localized on chromosome 9, close to the marker Z8363 [38]. The fact that some metaphases, in our study, showed four signals may be due to the presence of two *MSTN* genes in *S. aurata*. The relative similarity at the nucleotide level of the two myostatin cDNA genes (72%) could be enough to find some metaphase spreads with four signals. In the light of the results, the two genes (*MSTN-1* and *MSTN-2*) are located on different chromosomes, as was found in zebrafish, where the *MSTN-2* gene was situated on chromosome 22 (Zv7 of the zebrafish genome, NCBI data base). Our results show that the genotypes of *MSTN-1* gene should be considered for selective improvement programmes of *S. aurata *and could be a start point in order to detect candidate genes for growth in other aquatic species of economic interest.

## Figures and Tables

**Table 1:** Description of RFLPs in five candidate genes related to growth traits in gilthead seabream analyzed in this work.

Gene	PCR fragment	Enzyme used to detect polymorphism	Polymorphic region	RFLP alleles
*GH*	A: Exon 1 to intron2	*Hae*III &* Hap*II	Intron I	Size (micros.)
*GH*	B: Exon 4 to 3′UTR	*Hae*III, *Mbo*II, *Pst*I & *Taq*I	N.P.	—
*IGF-I*	A: Promoter to exon 1	*Taq*I	Promoter	T→G/C
*IGF-I*	B: Exon 1 to exon 2	*Hae*III, *EcoR*I *Dra*I, *Hap*II & *BspLU11*I	N.P.	—
*MSTN-1*	A: Promoter to exon 2	*Hae*III	Exon 1	C→T
		*Dra*I	Intron 1	A→C
*MSTN-1*	B: Exon 2 to exon 3	*Hae*III	Intron 2	G→T
*PRL*	A: Promoter to exon 1	*SspB*I & *Dra*I	Promoter	Size (micros.)
*PRL*	B: Exon 1 to exon 2	*EcoR*I	Intron 1	A→G
		*BspLU11*I	Intron 1	C→T
*PRL*	C: Exon 3 to exon 5	*Hae*III	Intron 3/Exon 4	Size (indel)
		*Fok*I	Intron 3 & 4/Exon 3	Size (indel)
*SL*	A: Promoter to exon 2	*Hae*III, *Hap*II, *Afa*I, *Dra*I & *Taq*I	N.P.	—
*SL*	B: Exon 4 to exon 5	*Tru9*I, *Taq*I, *Ssp*I, *Hae*III	N.P.	—

N.P.: nonpolymorphic; *GH*: growth hormone; *IGF-1*: insulin-like growth factor I; *MSTN*: myostatin; *PRL*: prolactin; *SL*: somatolactin.

**Table 2:** PCR oligonucleotide primers used for RFLP analysis in the five candidate genes studied in gilthead seabream.

Amplified fragment		Primers			
Fragment	Length (bp)	Name	Position	Sequence (5′-3′)	Tm (°C)
*GH_A*	1045–1900	SG1 [20]	Exon 1	AGAACCTGAACCAGACATGG	52.8
		*R1R1* [20]	Intron 2	CTGCTGCCAGAGAATTACTG	54.1
*GH_B*	1172	*GH*_4F	Exon 4	GTTCTCTGTCTGGCGGTTCT	56.3
		*GH*_4R	3′UTR	AGCAACTGGGTCTAATGAATGT	54.4
*IGF-I_A*	944/954	*IGFI_PF*	Promoter	TCATCAGATGTCATTTGCAGAC	52.9
		*IGFI_PR*	Exon 1	TGCCACTGAAAGGAAAGAGC	56.8
*IGF-I_B*	1506	*IGFI-1F*	Exon 1	AGCGCTCTTTCCTTTCAGTG	56.8
		*IGFI-1R*	Exon 2	GCCTCTCTCTCCACACACAA	55.1
*MSTN1_*A	934	*MSTN_1F*	Promoter	GCCTGTCAGTGTGGGACTTT	55.0
		*MSTN_1R*	Exon 2	ACGATTCGATTGGCTTGAAT	57.4
*MSTN1_B*	881/886	*MSTN_2F*	Exon 2	AACAAGTGTTGAGCGTGTGG	55.5
		*MSTN_2R*	Exon 3	CTCCGAGATCTTCACCTCCA	57.2
*PRL_A*	1014–1063	*PRL_PF *	Promoter	GACTTTAACATGACCTGGAGGA	54.4
		*PRL_PR*	Exon 1	TGGTTTCTCTGTGAGCCATCT	57.7
*PRL_B*	864/765	*PRL_1F*	Exon 1	ATGGCTCACAGAGAAACCAA	54.0
		*PRL_1R*	Exon 2	GAGTTGTGCTGAGGGAGTGC	55.7
*PRL_C*	1549/1496	*PRL_3-4F*	Exon 3	GTAGGCTGGACGATGATGC	54.8
		*PRL_3-4R*	Exon 5	AGCAGGACAACAGGAAATGG	56.7
*SL_A*	1650	*SL_PF*	Promoter	GAACAGTGGTAATGACAGTCTCAA	53.9
		*SL_PR*	Exon 2	GTGAGCAGATAGGGCCAGAG	56.2
*SL_B*	704–721	*SL_4F*	Exon 4	CATTCTGTGCTGATGCTGGT	55.9
		*SL_4R*	Exon 5	GGGCATCTTTCTTGAAGCAG	56.1

N.P.: nonpolymorphic; *GH*: Growth Hormone; *IGF-1*: Insulin-Like Growth Factor I; *MSTN*: Myostatin; *PRL*: Prolactin; *SL*: Somatolactin. A, B, and C fragments defined by the primer positions.

**Table 3:** Means (standard deviation) for body weight, fork length, and condition index in samples analyzed.

Group	*N*	Weight (g)	Length (cm)	Condition index (g · cm^−3^)
B-M	48	2,048.42 (±533.75)	50.97 (±3.85)	1.50 (±0.13)
B-F	83	2,656.22 (±491.51)	55.86 (±3.00)	1.52 (±0.15)
B-T	131	2,433.51 (±584.64)	54.07 (±4.08)	1.51 (±0.14)
J120	148	6.14 (±3.62)	7.22 (±1.51)	1.43 (±0.26)
A635	108	394.44 g (±139.56)	27.73 (±3.13)	1.77 (±0.17)
A819	120	549.58 g (±183.14)	29.14 (±2.87)	2.13 (±0.20)

B-M, B-F, and B-T: males, females, and total individuals among breeders, respectively; J120: juveniles sampled 120 days after hatching; A635 and A819: adults sampled 635 and 819 days after hatching.

**Table 4:** Analysis of variance (ANOVA) for weight, length, and condition index within genotypes for different age groups in *S. aurata. *Breeders are considered without respect to gender.

Locus		Phenotypic variable												
			*W*-B	*L*-B	*C*-B	*W*-J120	*L*-J120	*C*-J120	*W*-A635	*L*-A635	*C*-A635	*W*-A819	*L*-A819	*C*-A819
*IGF-I-P*	*Taq*I	*F*	0.325	0.492	0.758	0.046	0.243	4.227	3.328	3.101	1.227	1.457	1.408	1.137
		*P*	0.807	0.689	0.520	0.996	0.913	0.003^b^	0.040^a^	0.049^a^	0.297	0.220	0.236	0.343
		*N*	131	131	131	148	148	148	108	108	108	120	120	120

*MSTN1_A*	*Hae*III	*F*	8.010	8.578	0.614	0.177	0.105	0.117	0.665	0.807	0.297	3.521	2.832	2.546
		*P*	0.005^b^	0.004^b^	0.435	0.675	0.675	0.732	0.417	0.371	0.587	0.063	0.095	0.113
		*N*	131	131	131	148	148	148	108	108	108	120	120	120
	*Dra*I	*F*	0.303	0.712	0.290	0.012	0.003	1.430	0.422	1.859	11.918	—	—	—
		*P*	0.583	0.400	0.591	0.912	0.960	0.234	0.518	0.176	0.001^b^	—	—	—
		*N*	131	131	131	148	148	148	108	108	108	120	120	120

*MSTN1_B*	*Hae*III	*F*	4.236	3.723	0.440	5.103	3.924	0.047	4.747	3.292	4.510	0.518	0.144	2.929
		*P*	0.017^a^	0.027^a^	0.645	0.007^b^	0.022^a^	0.954	0.011^a^	0.041^a^	0.013^a^	0.597	0.866	0.057
		*N*	131	131	131	148	148	148	108	108	108	118	118	118

*PRL_1*	*Eco*RV	*F*	1.534	1.779	0.416	0.182	0.136	1.255	1.587	1.380	3.429	2.111	2.043	0.573
		*P*	0.220	0.173	0.660	0.834	0.873	0.288	0.197	0.253	0.020^a^	0.126	0.134	0.565
		*N*	131	131	131	148	148	148	108	108	108	120	120	120
	*BspLU*11I	*F*	1.123	1.199	0.415	0.453	0.463	0.716	2.086	1.882	1.513	0.944	1.019	0.365
		*P*	0.352	0.313	0.838	0.810	0.803	0.613	0.061	0.102	0.181	0.467	0.417	0.900
		*N*	131	131	131	148	148	148	108	108	108	120	120	120

*PRL_3_4*	*Fok*I	*F*	0.030	0.026	0.103	1.022	1.395	0.400	0.437	0.122	1.230	0.416	0.336	0.653
		*P*	0.97	0.974	0.902	0.362	0.251	0.671	0.647	0.885	0.297	0.661	0.715	0.522
		*N*	131	131	131	147	147	147	107	107	108	119	119	119

*W*-, *L*-, and *C*-: body weight, fork length, and condition index, respectively; B: breeders; J120: juveniles sampled 120 days after hatching; A635: adults sampled 635 days after hatching; A819 Adults sampled 819 days after hatching; *F*: *F* value; *P*: *P* value; *N*: number of individuals analyzed.

^a^Significant at *α* = 0.05.

^b^Significant at *α*_Bonferroni_ = 0.00714.

**Table 5:** Probability values (*P*) for exact tests for homogeneity of RFLP genotype frequencies in candidate genes between analyzed groups of *S. aurata. *

Locus			B	J-S			J-L			A635	A819
			F-M	S-M	M-L	S-L	S-M	M-L	S-L	S-L	S-L
*IGF-I-P*	*Taq*I	*P*	0.623	1.000	0.772	1.000	0.547	0.576	0.435	0.334	0.019^a^
		*N*	131	50	51	51	48	47	49	108	120

*MSTN1_A*	*Hae*III	*P*	0.157	0.349	0.191	1.000	1.000	1.000	1.000	0.617	0.119
		*N*	131	50	51	51	48	47	49	108	120
	*Dra*I	*P*	0.355	0.609	0.349	1.000	1.000	1.000	1.000	1.000	—
		*N*	131	50	51	51	48	47	49	108	120

*MSTN1_B*	*Hae*III	*P*	0.309	0.616	0.005^a^	0.002^b^	0.817	0.442	0.324	0.017^a^	1.000
		*N*	131	50	51	51	48	47	49	108	120

*PRL_1*	*Eco*RV	*P*	0.144	0.566	0.248	0.727	0.725	1.000	1.000	0.327	0.092
		*N*	131	50	51	51	48	47	49	108	120
	*BspLU*11I	*P*	0.281	0.443	0.404	0.762	1.000	0.732	0.719	0.054	0.196
		*N*	131	50	51	51	48	47	49	108	120

*PRL_3_4*	*Fok*I	*P*	0.889	1.000	0.427	0.313	1.000	0.818	1.000	0.788	0.407
		*N*	131	50	51	51	48	47	49	108	119

B: breeders; J: juveniles; A635: adults sampled 635 days after hatching; A819: adults sampled 819 days after hatching; S: small; M: medium; L: large; *N*: number of samples analyzed; *P*: *P* value.

^a^Significant at *α* = 0.05.

^b^Significant at *α*_Bonferroni_ = 0.0024.
